# Medical News Items

**Published:** 1864-08

**Authors:** 


### MEDICAL NEWS ITEMS.

Prof. Flint is about to publish a work on practical medicine.——Prof. Weber, of Cleveland, O., is about to sail for Europe on business connected with the opening of a new school in that city.——The telegraph suture, introduced by Mr. Clover, consists of a fine copper wire covered with gutta-percha, hence its name. It has been employed as an intercepted suture in hare-lip, leaving less scar than hare-lip pin. It is very pliable, can be knotted, and is as readily taken out as the silk ligature.——Prof. Pope, of St. Louis, recently removed a fœtal skeleton of extra-uterine formation through the rectum, and the patient recovered.——Prof. Chapman, of the Long Island College Hospital, received from the class an elegant gold chain.—— An abridgment of Copeland's Medical Dictionary, brought down to the present time, is announced as in course of preparation under the supevision of its author.——Prof. Langenbeck has been appointed Surgeon-General of the Prussian army.——Prof. Skoda has received the Grand Cross of the Guadaloupe Order from the Emperor Maximilian I.——Dr. Alfred Steele has been elected Professor of the Practice of Medicine in the University of Penn., in place of Prof. Pepper.——Dr. B. Howard Rand has been elected to the Chair of Chemistry in the Jefferson Medical College in place of Prof Bache.—*Am. Medical Times.*

